# Colon-Specific Delivery of Probenecid Enhances Therapeutic Activity of the Uricosuric Agent Against Rat Colitis

**DOI:** 10.3390/pharmaceutics17111454

**Published:** 2025-11-11

**Authors:** Yeonhee Jeong, Jaejeong Kim, Changyu Kang, Yunjin Jung

**Affiliations:** 1 College of Pharmacy, Pusan National University, Busan 46241, Republic of Korea; jyhsyh0518@naver.com (Y.J.); wowjd9669@naver.com (J.K.); whale10000@naver.com (C.K.); 2 Research Institute for Drug Development, Pusan National University, Busan 46241, Republic of Korea

**Keywords:** probenecid, colon-targeted drug delivery, colitis, prodrug

## Abstract

**Background/Objectives**: Probenecid (PBN) is a uricosuric agent that facilitates the excretion of uric acid and is used to treat gout. Here, a colon-targeted prodrug of PBN was designed to facilitate repositioning as a treatment for inflammatory bowel disease (IBD). **Methods**: The carboxylic group in PBN was amide-conjugated with the amine groups of acidic amino acids to yield aspartic acid-conjugated PBN (PBN-AA) and glutamic acid-conjugated PBN (PBN-GA). Conjugation with amino acids increased the hydrophilicity of PBN and decreased cell permeability across the Caco-2 cell monolayer. While remaining intact in buffers (pH 1.2, 6.8) and in the small intestinal contents of rats, the conjugates were cleaved to release PBN from the cecal contents of rats, with a significant difference in the maximal conversion percentage between PBN-AA (12%) and PBN-GA (74%). **Results**: Upon oral gavage, PBN-GA accumulated a much greater amount of PBN in the cecum than PBN alone, thus verifying the in vitro colon specificity of PBN-GA. Oral PBN-GA enhanced the anticolitis effectiveness in dinitrobenzene sulfonic acid-induced rat colitis and limited the systemic absorption of PBN, thus reducing the risk of systemic adverse effects ascribed to PBN. Moreover, PBN-GA therapeutically surpassed sulfasalazine, a currently used anti-IBD drug, in rat colitis. **Conclusions**: These results suggest that amide conjugation with GA can be used to design a colon-targeting prodrug for PBN. Colon-targeted PBN may not only enhance therapeutic effectiveness but also improve the safety of PBN repositioned for the treatment of IBD and may be a pharmacological alternative for current small-molecule anti-IBD drugs with low efficacy or serious adverse effects with long-term use.

## 1. Introduction

Inflammatory bowel disease (IBD), which includes ulcerative colitis (UC) and Crohn’s disease (CD), is a chronic, pharmacologically incurable, and relapsing inflammatory disorder that occurs largely in the distal part of the gastrointestinal tract (GIT) [1,2]. Despite extensive genetic and biological research on IBD over the past decades, its exact pathogenesis remains unclear.

Anti-IBD drug therapies require lifelong administration and aim to induce and maintain remission of inflammation [3]. Conventional drug therapies, including mesalazine (5-ASA), glucocorticoids, and immunosuppressants such as thiopurines and methotrexate, are not satisfactory because of their low anti-inflammatory efficacy, serious adverse effects with long-term treatment, and the development of treatment refraction over time [4,5]. Recently, biologics comprising anti-tumor necrosis factor, anti-α_4_β_7_ integrin, anti-interleukin (IL)-12, and IL-23 agents, and small-molecule drugs such as Janus kinase (JAK) inhibitors and sphingosine-1-phosphate (S1P) receptor modulators have been introduced as anti-IBD drugs, which has expanded the therapeutic landscape for IBD, especially for patients with severe IBD [6]. Clinically, these drugs elicit superior therapeutic outcomes in patients with IBD refractory to conventional drug therapies. However, new anti-IBD drugs still have medical and practical drawbacks due to therapeutic tolerance ascribed to the production of antibodies against biologics, adverse effects resulting from long-term therapy, medication costs, and poor patient compliance due to parenteral administration [5,6,7]. Given the drawbacks of current drug therapies, the development of small-molecule anti-IBD drugs with enhanced efficacy and safety, applicable to patients with moderate to severe IBD, is still warranted.

Probenecid (PBN), originally developed to extend the half-life of antibiotics such as penicillin by retarding kidney excretion, has been used for the treatment of gout caused by high blood uric acid levels for several decades [8]. It facilitates the excretion of uric acid by inhibiting its reabsorption into the kidneys. Recently, PBN has drawn interest as a candidate drug for repositioning for medical applications, including IBD, autoimmune diseases, neuroinflammation, and cardiovascular diseases, based on the finding that PBN inhibits pannexin-1 (Px1) channels [9,10,11,12]. Px1 channels are principal exporters of ATP and mediate the activation of caspase-1 and release of IL-1β induced by P2X7 receptor activation [13,14]. In various cell types, including neurons and macrophages, PBN inhibits inflammasome activity, probably by blocking Px1 channels [9,15,16]. A Px1 inhibitor effectively protects the human colonic mucosal barrier against cytokine-induced colitis [17]. In addition, Px1 inhibitors, including PBN, prevent the death of enteric neurons caused by intestinal inflammation in IBD, thereby preserving the functional control of the colonic musculature [18].

Despite the therapeutic potential of the FDA-approved drug PBN for the treatment of IBD, no prior study has tested its anti-IBD potential. In the present study, we investigated whether PBN had potential as an anti-IBD agent. Colon-targeted drug delivery (CTDD) has been applied to PBN to enhance its value as an anti-IBD drug. In CTDD, the orally administered drug is delivered to the large intestine without significant loss in the stomach and small intestine [19]. CTDD can be accomplished using both pharmaceutical and prodrug approaches [20]. The pharmaceutical approach employs formulation techniques such as pH-sensitive or time-dependent coating, while the prodrug approach develops pharmacologically inactive drug derivatives that exhibit minimal systemic absorption and presystemic metabolism and are readily converted to the active parent compound in the large intestine [19]. In general, CTDD enhances drug availability within the large intestine, while simultaneously minimizing systemic absorption [20]. Therefore, this delivery strategy has the potential to enhance both the therapeutic efficacy and safety of drugs used for the treatment of colonic disorders such as IBD [21,22,23,24,25]. Previous studies have demonstrated that CTDD can facilitate the repositioning of drugs such as riluzole and sofalcone as anticolitic agents by improving their therapeutic profiles [23,26].

In this study, colon-targeted prodrugs of PBN were synthesized and their colon specificity was assessed via in vitro and in vivo experiments. The anti-colitis effects of a colon-targeted prodrug of PBN showing better colon-specific performance were assessed in a dinitrobenzene sulfonic acid (DNBS)-induced rat colitis model and compared with those of PBN and sulfasalazine (SSZ), a clinically available anti-IBD prodrug of 5-ASA [4,27]. To evaluate whether colon-targeted PBN improved safety, the systemic absorption of PBN was assessed by measuring the drug in the blood of rats after oral administration of PBN and colon-targeted PBN.

## 2. Materials and Methods

### 2.1. Materials

PBN and DNBS were sourced from Tokyo Chemical Industry (Tokyo, Japan) and SSZ was obtained from Sigma-Aldrich Co. (St. Louis, MO, USA). *L*-Aspartic acid dimethyl ester (AA-DME) hydrochloride, *L*-glutamic acid dimethyl ester (GA-DME) hydrochloride, and 1,1′-carbonyldiimidazole (CDI) were purchased from Ambeed, Inc. (Arlington Heights, IL, USA). Organic solvents for the reactions and analytical-grade high-performance liquid chromatography (HPLC) solvents were supplied by Junsei Chemical Co. (Tokyo, Japan) and DAEJUNG Chemicals & Metals Co., Ltd. (Gyeonggi-do, Republic of Korea), respectively. The enzyme-linked immunosorbent assay (ELISA) kit for cytokine-induced neutrophil chemoattractant-3 (CINC-3) was obtained from R&D Systems (Minneapolis, MN, USA). Phosphate-buffered saline (PBS; pH 7.4) was obtained from Thermo Fisher Scientific (Waltham, MA, USA). All additional chemicals used were of reagent grade and commercially available. Visualization of spots on silica gel TLC plates (F2545, Merck Millipore, Burlington, MA, USA) was performed using a 254 nm UV lamp. IR and ^1^H-NMR spectra were recorded using a Nicolet iS50 FT-IR spectrophotometer (Thermo Fisher Scientific, Waltham, MA, USA) and a Varian ^1^H-NMR spectrometer (Palo Alto, CA, USA), respectively. NMR chemical shifts were reported in ppm relative to those of tetramethylsilane. Electrospray ionization mass spectrometry (ESI-MS) was performed using an Agilent 65,360 Q-TOF mass spectrometer (Agilent, Santa Clara, CA, USA).

### 2.2. Synthesis of PBN Derivatives

To activate the carboxylic group of PBN (285.3 mg, 1 mmol), it was reacted with CDI (201.7 mg, 1.3 mmol) in 20 mL of acetonitrile (ACN) for 2 h. GA-DME hydrochloride (317.4 mg, 1.5 mmol) and trimethylamine (TEA, 1 mL) were added to the reaction mixture, which was then stirred at 20–25 °C for 48 h. The solvent was removed under reduced pressure and the residue was dissolved in ethyl acetate (EA). The organic phase was washed thrice with 0.5 M hydrochloric acid and 5% NaHCO_3_. After evaporation of the solvent, the remaining residue was treated with 30 mL of 0.5 M NaOH and stirred at 20–25 °C for 2 h, followed by acidification using 1.0 M hydrochloric acid and subsequent extraction with EA. The organic layer was dried over NaSO_4_, filtered, and removed to obtain PBN-GA as a white powder. PBN-GA was recrystallized with ether and n-hexane (1:9 *v*/*v*). PBN-GA yield was 28%; mp: 262 °C (decomp); FT-IR (ATR), ν_max_ (cm^−1^): 1711 (C=O, carboxyl), 1639 (C=O, amide); ^1^H-NMR (500 MHz, DMSO-d6): δ 8.06 (d, *J* = 8.5 Hz, 2H), 7.91 (d, *J* = 8.5 Hz, 2H), 4.42 (m, 1H), 3.06 (dd, *J* = 16.6, 9.1 Hz, 4H), 2.38 (t, *J* = 7.5 Hz, 2H), 2.13 (m, 1H), 1.97 (m, 1H), 1.47 (m, 4H), 0.82 (t, *J* = 7.4 Hz, 6H). ESI-MS [M-*H*]^−^: *m*/*z* = 413.1403.

PBN-AA was synthesized using AA-DME hydrochloride using the same method as for PBN-GA. PBN-AA yield was 31%; mp: 258 °C (decomp); FT-IR (ATR), ν_max_ (cm^−1^): 1715 (C=O, carboxyl), 1643 (C=O, amide); ^1^H-NMR (500 MHz, DMSO-d6): δ 8.02 (d, *J* = 8.5 Hz, 2H), 7.92 (d, *J* = 8.5 Hz, 2H), 4.77 (m, 1H), 3.07 (m, 4H), 2.86 (dd, *J* = 16.5, 5.6 Hz, 1H), 2.73 (dd, *J* = 16.5, 8.3 Hz, 1H), 1.47 (m, 4H), 0.81 (t, *J* = 7.4 Hz, 6H). ESI-MS [M-*H*]^−^: *m*/*z* = 399.123

### 2.3. HPLC Analysis

A Symmetry C18 column (Hector, Theale, Berkshire, UK; 250 × 4.6 mm, 5 μm) connected to a HPLC system (Gilson, Middleton, WI, USA) was used for chromatographic separation of samples. Mobile phases A (5:2.5:2.5, *v*/*v*) and B (6:2:2, *v*/*v*) consisted of 1.0 mM sodium phosphate buffer with 0.5 mM tetrabutylammonium chloride, ACN, and MeOH. The HPLC analysis was conducted at a flow rate of 1 mL/min. The eluate was monitored at 247 nm using a UV detector to measure the absorption and had a sensitivity of AUFS 0.01. The retention times of PBN (mobile phase A), PBN-GA, and PBN-AA (mobile phase B) were 5.1, 9.9, and 11.2 min, respectively.

### 2.4. Distribution Coefficient and Chemical Stability

Distribution coefficients (log *D*_6.8_) of PBN, PBN-GA, and PBN-AA were measured using an isotonic phosphate buffer (pH 6.8)/n-octanol system, as described previously [22].

The chemical stabilities of PBN-GA and PBN-AA were tested in HCl-NaCl buffer (pH 1.2) and isotonic phosphate buffer (pH 6.8). Changes in the concentrations of PBN-GA and PBN-AA were monitored by HPLC.

### 2.5. Cell Permeability Assay

The cell permeabilities of PBN, PBN-GA, and PBN-AA were evaluated using a Caco-2 cell monolayer model. Caco-2 cells were seeded at a density of 4 × 10^5^ cells per insert in Transwell 6-well plates with a pore size of 0.4 μm (SPL Life Sciences, Pocheon, Gyeonggi-do, Republic of Korea). The cells were cultured in Dulbecco’s modified eagle medium (DMEM) supplemented with 10% Fetal bovine serum (FBS) and 1% penicillin/streptomycin until the transepithelial electrical resistance (TEER) measured using an EMD Millipore system (Billerica, MA, USA) reached 2000 Ω·cm^2^, indicating an intact monolayer.

For the transport study, the apical (donor) compartment was filled with 2 mL of DMEM without phenol red containing the test compounds (500 μM), and 3 mL of DMEM without phenol red was placed in the basolateral (acceptor) compartment. Samples were collected from the basolateral side at designated time intervals up to 24 h to determine the concentration of the permeated compounds using HPLC. Each experiment was performed in triplicate, and monolayer integrity was confirmed by maintaining TEER values above 2000 Ω·cm^2^ before and after the assay [28].

### 2.6. Animals

Seven-week-old male Sprague–Dawley (SpD) rats were obtained from Samtako Bio Korea (Gyeonggi-do, Republic of Korea) and maintained under controlled environmental conditions in an animal facility at Pusan National University (Busan, Republic of Korea). All animal procedures complied with the ARRIVE guidelines and were approved by the Institutional Animal Care and Use Committee of Pusan National University (PNU–IACUC) in accordance with the ethical guidelines for animal research (Approval No: PNU-2024-0229, Approval Date: 12 October 2024).

### 2.7. Incubation of Drugs in the Contents of Rat Small Intestine and Cecum

Male Sprague-Dawley rats (250–260 g) were euthanized with CO_2_ prior to making a midline abdominal incision. The small intestinal contents were collected and suspended in isotonic phosphate buffer (pH 6.8) to prepare a 20% (*w*/*v*) suspension. PBN-GA (2 mM) was dissolved in 3 mL of isotonic phosphate buffer (pH 6.8), were mixed with 3 mL of the small intestinal suspension and incubated at 37 °C. A solution of PBN-AA was created with the same method. At designated time points, 0.5 mL of the reaction mixture was collected and centrifuged at 10,000× *g* at 4 °C for 10 min, and 0.1 mL of the supernatant was mixed with 0.9 mL of methanol. After vortexing and centrifugation under the same conditions, the final supernatant was filtered through a 0.45 μm membrane, and 20 μL of the filtrate was analyzed for PBN concentration by HPLC.

The cecal contents were collected and suspended in isotonic phosphate buffer (pH 6.8) to prepare a 20% (*w*/*v*) suspension. To maintain anaerobic conditions, the suspension was handled under a nitrogen atmosphere using an anaerobic chamber bag (AtmosBag, Thermo Fisher Scientific, Waltham, MA, USA). Incubation with PBN-GA and PBN-AA, sample collection, and HPLC analysis were performed under the same conditions as those described for the small intestinal suspensions.

### 2.8. Analysis of Drug Concentration in Blood and Cecum

Male Sprague–Dawley rats had food withheld for 24 h but had access to water. The rats (*n* = 30) were randomly divided into 6 groups (*n* = 5 per group) and treated with either PBN (100.0 mg/kg) or PBN-GA (145.4 mg/kg, equivalent to 100.0 mg/kg PBN) in 1.0 mL PBS via oral gavage. Blood samples were collected via cardiac puncture at 2, 4, and 8 h post-administration and immediately centrifuged at 10,000× *g* for 10 min at 4 °C. A 0.1 mL aliquot of plasma was mixed with 0.9 mL of methanol, vortexed thoroughly, and centrifuged under identical conditions. The resulting supernatant was filtered through a 0.45 μm membrane, and 20.0 μL of the filtrate was injected into the HPLC system to determine plasma PBN concentrations.

For cecal distribution studies, rats were orally administered PBN (30.0 mg/kg) or PBN-GA (43.6 mg/kg, equivalent to 30.0 mg/kg of PBN) in 1.0 mL PBS. At 2, 4, and 8 h post-dosing, the cecal contents were harvested and suspended in isotonic phosphate buffer (pH 6.8) to prepare 10% (*w*/*v*) homogenates. Quantitative analysis of PBN in the cecal suspensions was performed using HPLC, following the procedure described in Section 2.7.

### 2.9. DNBS-Induced Rat Colitis

Experimental colitis was induced in rats as previously described [21]. Briefly, male Sprague–Dawley rats underwent to a 24 h fasting period with free access to water. Anesthesia was induced using 3% isoflurane and maintained at 2.5% in 1 L/min oxygen using a Small Animal O_2_ Single Flow Anesthesia System (LMS, Pyeongtaek, Republic of Korea). Once the animals no longer reacted to external tactile stimulation, a rubber cannula (2 mm outer diameter) was carefully inserted rectally, positioning the tip approximately 8 cm from the anus to target the splenic flexure. A solution of DNBS (48.0 mg) in 0.4 mL of 50% aqueous ethanol was then instilled into the colon via cannula to induce colitis.

### 2.10. Evaluation of Anticolitis Effects

Three days after the induction of colonic inflammation, the respective drugs were orally administered to the rats once daily for 6 consecutive days. The experimental scheme and treatment group assignments are shown in Supplementary Material S1. Colonic damage scores (CDS) were determined based on previously established scoring criteria [21]. The scoring system is shown in Supplementary Material S2. Three independent observers blinded to the treatment groups conducted the CDS assessments. Myeloperoxidase (MPO) activity in the distal colon (4 cm) was determined as previously [29]. One unit of MPO activity was defined as 1.0 μmol min^−1^ of peroxide degradation at 25 °C.

### 2.11. Western Blot Analysis and ELISA for CINC-3

Western blot analysis was performed on lysates derived from distal colon tissues following a previously established protocol [28]. The detailed procedure is shown in Supplementary Material S3. Western blot images were quantified using Image Lab software (version 5.2 build 14; Bio-Rad, Hercules, CA, USA). Results are presented as the mean of the quantified values for each Western blot in the figures (*n* = 5).

The levels of inflammatory cytokine-induced neutrophil chemoattractant-3 (CINC-3) in the inflamed distal colon were determined using a CINC-3 enzyme-linked immunosorbent assay (ELISA) kit (R&D Systems, Minneapolis, MN, USA) as previously described [28].

### 2.12. Data Analysis

Data are presented as mean values with standard deviations (mean ± SD). Statistical differences between groups were evaluated using one-way analysis of variance (ANOVA), followed by Tukey’s post hoc test. The Mann–Whitney U test was used for comparisons of CDS. Differences were considered statistically significant at *p* < 0.05.

## 3. Results

### 3.1. Synthesis of Probenecid Conjugated with Acidic Amino Acids

**Figure 1:**
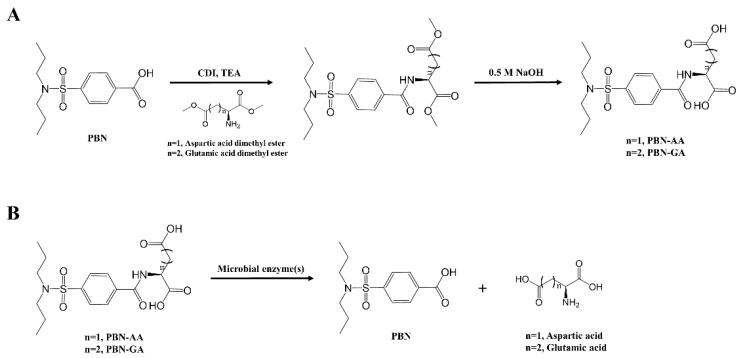

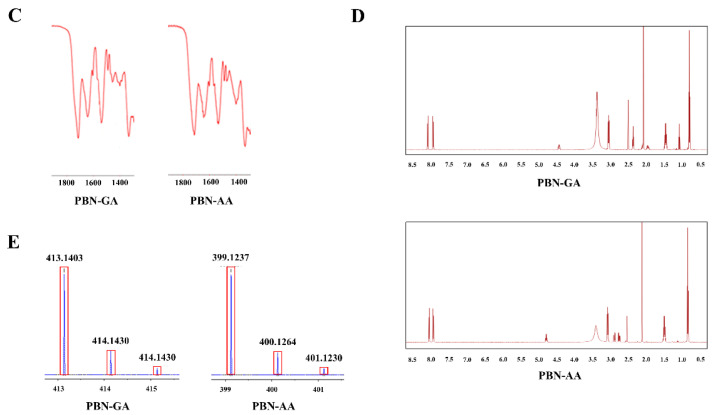
Synthesis and spectroscopic characterization of PBN derivatives (**A**) Synthesis of PBN derivatives, (**B**) Proposed pathway for colonic activation of PBN derivatives, (**C**) FT-IR spectra of PBN derivatives, (**D**) ^1^H-NMR spectra of PBN derivatives, and (**E**) Mass spectra of PBN derivatives.

To impose colon-specificity on PBN, the amino groups in the acidic amino acids GA and AA were conjugated with the carboxylic group in PBN. The synthetic scheme and the proposed colonic activation of the conjugates are shown in Figure 1A,B. The synthesis of the final products, PBN-GA and PBN-AA, was verified by instrumental analysis using FT-IR, ^1^H-NMR, and mass spectrometry. As shown in Figure 1C, the amide bonds formed by the conjugation of PBN and amino acids were observed at 1639 cm^−1^ (for PBN-GA) and 1643 cm^−1^ (for PBN-AA). In ^1^H-NMR spectra, proton peaks ascribed to amino acids and PBN were clearly detected (Figure 1D). Mass spectrometry revealed molecular peaks corresponding to PBN-GA and PBN-AA (Figure 1E). The original spectra are shown in Supplementary Material S4.

### 3.2. Colon Specificity of PBN Conjugated with Acidic Amino Acids

**Figure 2:**
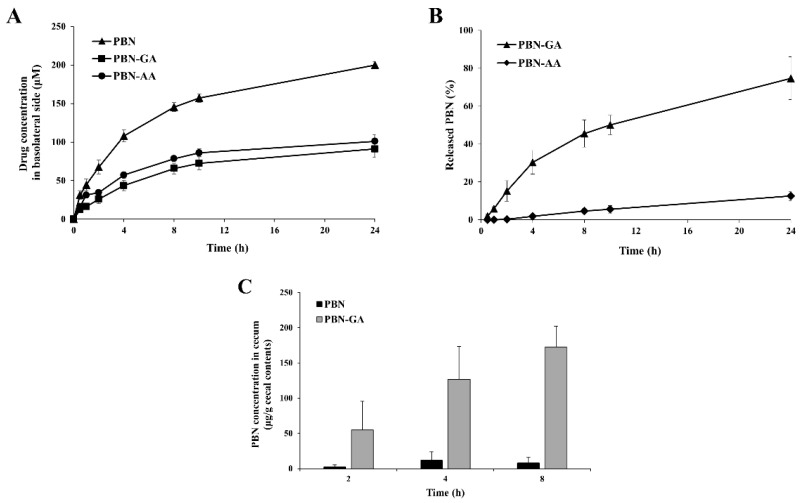
PBN derivatives are colon-specific prodrugs of PBN. (**A**) PBN and PBN derivatives (500 μM, 2.0 mL) dissolved in culture medium were applied to the apical side of the Caco-2 cell monolayer and incubated for 24 h. At predetermined time points, the concentrations of each compound in the basolateral compartment (3 mL) were quantified by HPLC. (**B**) PBN derivatives (2.0 mM) were incubated with the cecal contents suspended in isotonic phosphate buffer (pH 6.8, 10%) under nitrogen. The concentrations of PBN were analyzed using HPLC at appropriate time intervals. (**C**) Male SpD rats (250–260 g) underwent a 24 h fast while water was provided ad libitum. Rats were treated orally with PBN (30.0 mg/kg) and PBN-GA (43.6 mg/kg, equivalent to 30.0 mg/kg of PBN) suspended in PBS. The rats were euthanized at 2, 4, and 8 h after administration, and concentrations of PBN in the cecum were quantified using HPLC. The data are presented as mean ± SD (*n* = 5).

We examined whether the acidic amino acid-conjugated PBN, PBN-GA, and PBN-AA were colon-specific. For the conjugates to be colon-specific, orally administered conjugates must remain intact and not significantly absorbed as they pass through the stomach and small intestine and subsequently undergo bioconversion to PBN upon reaching the large intestine. Therefore, we first tested whether the conjugates were stable during incubation in buffers with pH 1.2 and 6.8, representing the luminal pH of the stomach and small intestine, and in the small intestinal contents of rats. Under these conditions, there was no significant change in the concentration of the conjugates after 10 h. To assess whether the amino acid conjugation of PBN limited the systemic absorption of PBN, distribution coefficients (log *D*_6.8_) and cell permeability were measured using Caco-2 cell monolayers. The amino acid conjugation lowered log *D*_6.8_ of PBN (0.3) to −1.22 (PBN-GA) and −1.04 (for PBN-AA). In parallel, amino acid-conjugated PBN exhibited lower transport via the cell monolayer than PBN (Figure 2A). Conjugates delivered to the large intestine must be converted to their parent drug, PBN, to meet colon-specific conditions. The conjugates were incubated in cecal contents under a nitrogen atmosphere in an anaerobic chamber bag to simulate the anaerobic conditions of the large intestine. As shown in Figure 2B, PBN-GA was gradually converted to PBN, reaching 45% at 8 h and 74% at 24 h. In contrast, the conversion of PBN-AA was significantly less efficient, with only 4% and 12% conversion observed at the same time points, respectively. These findings indicate that a substantial portion of orally administered conjugates reaches the large intestine without undergoing significant presystemic metabolism or systemic absorption, and that PBN-GA, but not PBN-AA, efficiently releases PBN in the colonic environment. To verify the colon specificity of PBN-GA, either PBN-GA or PBN was administered orally to rats, and the concentrations of PBN were determined in the cecum at 2, 4, and 8 h after administration. As shown in Figure 2C, consistent with the in vitro results, a much greater amount of PBN was detected in the cecum at all time points after oral PBN-GA administration, and the highest concentration of PBN was observed at 8 h, indicating that PBN-GA satisfied the colon-specific conditions, thus acting as a colon-targeted prodrug.

### 3.3. PBN-GA Enhances the Anticolitis Activity of PBN and Is Therapeutically Superior to SSZ

**Figure 3:**
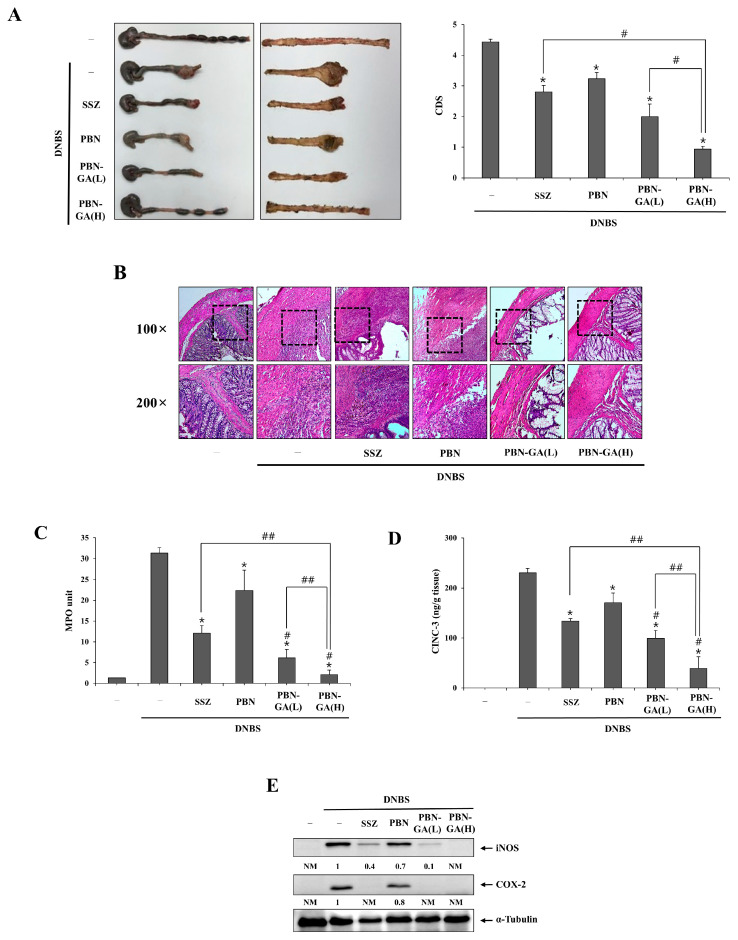
PBN-GA is therapeutically superior to PBN and SSZ in rat colitis. Three days following colitis induction, rats were orally administered SSZ (50.0 mg/kg), PBN (30.0 mg/kg), PBN-GA (L) (21.8 mg/kg, equivalent to 15 mg/kg of PBN), or PBN-GA (H) (43.6 mg/kg, equivalent to 30 mg/kg of PBN) each dissolved in 1.0 mL PBS (pH 7.4), once daily. Animals were sacrificed 24 h after the sixth dose. (**A**) Left panel: Representative photographs of the serosal and luminal surfaces of the distal colon. Right panel: Colonic damage score (CDS) for each treatment group. (**B**) Hematoxylin and eosin (H&E) staining was conducted on colonic tissue sections from the different treatment groups. Upper panel: Representative images of 100× magnification. Lower panel: Higher-magnification images (200×) of the dotted areas in the upper panel. (**C**) Myeloperoxidase (MPO) activity was assessed in the inflamed distal colons (4.0 cm). (**D**) Levels of CINC-3 in the inflamed colon were quantified using an ELISA kit. (**E**) Expression of iNOS and COX-2 in the inflamed colon was analyzed by Western blotting. α-Tubulin was used as a loading control to normalize iNOS and COX-2 levels. The data are presented as mean ± SD (*n* = 5). * *p* < 0.05, vs. DNBS control. ^#^ *p* < 0.05, vs. PBN. ^##^ *p* < 0.05. NM: not measurable.

We examined whether the colon-targeted delivery of PBN exerted anticolitis effects in rat colitis and enhanced the anticolitis activity of PBN. In addition, the anticolitis efficacy of colon-targeted PBN was compared with that of SSZ, a colon-targeted prodrug of 5-ASA. Rat colitis was induced by rectal instillation of DNBS via oral gavage 3 days after colitis induction. Doses of SSZ (50.0 mg/kg) and PBN (30.0 mg/kg) were decided based on previous papers [30,31] and human doses (500.0 mg–2000.0 mg/day) for clinical uses [32,33], respectively. PBN (30.0 mg/kg), PBN-GA (21.8 mg/kg, equivalent to 15.0 mg/kg of PBN), PBN-GA (43.6 mg/kg, equivalent to 30.0 mg/kg of PBN), and SSZ (50.0 mg/kg) were administered orally to rats once per day for 6 consecutive days. The rats were sacrificed 24 h after the last dose, and the anticolitis effects of the drugs were evaluated. As shown in Figure 3A, the colitis control without medication showed severe colonic tissue damage due to inflammation, with hemorrhagic ulcers, extensive defoliation of the mucosa, stricture, edema, and adhesion to neighboring organs, along with shortening of the large intestine, which corresponded to approximately a 4.5 CDS. All drugs showed anticolitis activity, with alleviated colonic tissue damage and inflammation. The colonic tissue improvement was greater with PBN-GA than with PBN, regardless of the dose. In addition, PBN-GA at low and high doses was more effective than SSZ in alleviating colonic tissue damage. Hematoxylin and eosin (H&E) staining of the inflamed colon showed that mucosal recovery with PBN-GA occurred in a dose-dependent manner and was more effective than PBN and SSZ (Figure 3B). As shown in Figure 3C–E, colonic inflammation was attenuated by all drugs. PBN-GA dose-dependently diminished myeloperoxidase (MPO) activity (Figure 3C) and the levels of inflammatory mediators, COX-2, iNOS (Figure 3E), and CINC-3 (Figure 3D), and was more effective in suppressing the inflammatory response than PBN and SSZ, regardless of the dose of PBN-GA. These results indicate that colon-targeted PBN potentiates the anticolitis activity of PBN and therapeutically surpasses SSZ in rat colitis. Original images of Western blot and bar graphs presenting quantified image intensity as mean ± SD are shown in Supplementary Material S5.

### 3.4. PBN-GA Reduces the Risk of Systemic Adverse Effects of PBN

**Figure 4:**
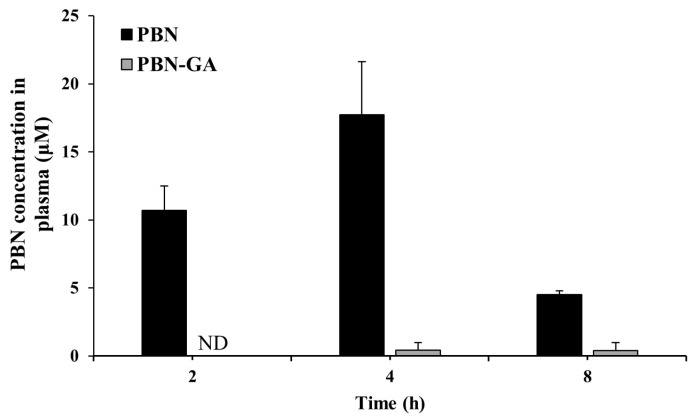
PBN-GA reduces systemic absorption of PBN. Male SpD rats (250–260 g) underwent a 24 h fast with free access to water. PBN (100.0 mg/kg) or PBN-GA (145.4 mg/kg, equivalent to 100.0 mg/kg of PBN), suspended in PBS (pH 7.4, 1 mL), was administered orally. Blood samples were obtained via cardiac puncture at 2, 4, and 8 h after administration. Plasma concentrations of PBN were determined using HPLC. The data are presented as mean ± SD (*n* = 5).

With drug repositioning, the original effect of a candidate drug becomes an adverse effect. Therefore, the repurposing of oncologic drugs for non-oncological use is not recommended. Moreover, it would be beneficial to minimize the original pharmacological activity of repositioned drugs, even for non-oncological drugs. To evaluate whether colon-targeted PBN can have improved safety with repositioning as an anti-IBD drug, concentrations of PBN were monitored in the blood at 2, 4, and 8 h after oral gavage of PBN (100.0 mg/kg) and PBN-GA (145.4 mg/kg equivalent to 100.0 mg/kg of PBN). As shown in Figure 4, oral PBN-GA had greatly reduced blood concentrations of PBN at all time points compared with oral PBN (1.4 μM–3.7 μM). The peak blood concentration of PBN obtained after oral administration of PBN-GA was lowered to approximately 1/45 of that obtained after the oral administration of PBN.

## 4. Discussion

IBD is an intractable chronic inflammatory disorder of the GI tract, and pharmacotherapy for IBD remains challenging despite the introduction of diverse anti-IBD biologics. Here, we show that colon-targeted PBN may repurpose PBN as an anti-IBD drug with enhanced therapeutic efficacy and safety and has potential as a pharmacological option to replace current small-molecule anti-IBD drugs whose clinical use is often limited by serious adverse effects and low efficacy [34].

PBN was chemically modified for colon specificity. Conjugation with the acidic amino acids AA and GA is a feasible method to create a colon-targeted prodrug of drugs such as PBN with a carboxylic group [35]. The amide bonds formed by the conjugation between the amine groups in the amino acids and the carboxylic group in the drug are stable in the host enzymes, whereas they are susceptible to microbial enzymes in the large intestine. In addition, the conjugation of the drug and amino acids creates conjugates possessing 2 carboxylic groups, resulting in higher hydrophilicity. For these reasons, the conjugates tend to remain intact during transit through the stomach and small intestine because of their poor passive transport across epithelial cell layers and biochemical stability against presystemic metabolism. Microbial enzymes in the large intestine readily cleave the conjugates to release the parent drug. Therefore, the designed conjugates satisfy the essential conditions for a colon-targeted prodrug to deliver a drug specifically to the large intestine without substantial loss in the upper intestine [19,35]. PBN conjugated with acidic amino acids are colon-specific, although their colon-specific performance is quite different. The PBN-acidic amino acid conjugates, PBN-GA and PBN-AA, lowered the distribution coefficient of PBN to −1.22 (for PBN-GA) and −1.04 (for PBN-AA) and significantly lowered the cell permeability of PBN across the Caco-2 cell monolayer, indicating that amino acid conjugation retarded the passive transport of PBN via the epithelial cell layer in the GI tract. While the conjugates of PBN were chemically and biochemically stable in the stomach and small intestine, PBN was liberated during incubation with cecal contents under anaerobic conditions. Notably, the rate of conjugate conversion to PBN varied by the amino acid used. PBN-GA was converted to PBN by up to 74%, whereas the conversion of PBN-AA was approximately 12%, suggesting different susceptibilities of the amide bonds of the conjugates to microbial enzymes. Colon specificity was verified in an animal experiment using PBN-GA with a higher conversion percentage. In agreement with the in vitro evaluation, the in vivo assessment found that more PBN was delivered and accumulated in the cecum with orally administered PBN-GA than with oral PBN at all time points. These in vitro and in vivo results indicated that PBN-GA is a colon-targeting prodrug of PBN. Colon-targeted PBN is likely to have enhanced therapeutic effectiveness upon repositioning as an anti-IBD drug. This is supported by the data showing that oral PBN-GA (even at low dose corresponding to 15.0 mg/kg of PBN) was more effective in alleviating colonic tissue damage and inflammation than oral PBN (30.0 mg/kg), strongly suggesting that PBN-GA potentiates the anticolitis effects of PBN. The enhanced anticolitis activity of PBN-GA may be associated with the colon-targeted delivery of PBN, resulting in an increase in PBN concentration at the inflamed site [20,36]. Compared to oral PBN, at least a ten-fold amount of PBN was observed in the large intestine at all time points after oral administration of PBN-GA. In support of this argument, significant inhibition of the potential target Px-1 for PBN-mediated anti-colitic effects likely requires a 1 mM concentration of PBN [11], which may not be achievable with oral PBN at human clinical doses. The concentration of PBN obtained in the cecum after oral PBN-GA reached up to approximately 0.6 millimole (PBN millimole/1 kg cecal contents). Molar concentration of PBN dissolved in the aqueous part may be higher than 0.6 mM due to low water content in the large intestine. Consistent with previous studies demonstrating that Px-1 inhibition suppresses the elevation of inflammatory mediators [37], the inflammatory mediators COX-2, iNOS, and CINC-3 were substantially downregulated by oral PBN-GA.

A study published in November 2025 demonstrated that GA administered orally at 100 mg/kg attenuates dextran sulfate sodium-induced colitis in mice [38], therefore the therapeutic contribution of GA to the anticolitis activity of PBN-GA should be considered, limiting the interpretation of the anticolitis performance of colon-targeted PBN. Further investigations are required to clarify the combined effects of colon-targeted PBN and GA.

Treatment of gout with PBN is usually continued indefinitely if it is effective and no serious adverse effects occur [8]. This is beneficial for repositioning PBN as an anti-IBD drug that requires long-term use [39,40]. Compared with PBN, PBN-GA reduced the systemic absorption of PBN, as shown by the substantially reduced blood concentrations of PBN, thus further alleviating the risk of systemic adverse effects of PBN.

## 5. Conclusions

Colon-targeted PBN, which enhances the anticolitis effectiveness and safety of PBN and therapeutically surpasses SSZ in rat colitis, may be a promising pharmacological option as a substitute for current small-molecule anti-IBD drugs that have limitations in clinical use because of their low anti-IBD efficacy and serious adverse effects.

## Data Availability

The data presented in this study are available in the article or Supplementary Materials.

## Supplementary Materials

The following supporting information can be downloaded at: https://www.mdpi.com/article/10.3390/pharmaceutics17111454/s1, S1: Animal experiments and treatment groups: (A) Detailed schemes of animal experiments, (B) Treatment groups in the animal experiments; S2: Modified scoring system; S3: Detailed protocol for Western blot; S4: Full spectra of PBN derivatives: (A) FT-IR spectra of PBN derivatives, (B) ^1^H-NMR spectra of PBN derivatives, (C) Mass spectra of PBN derivatives; S5: Original and quantified images of Western blot: (A) Original images of Western blot, (B) Bar graphs presenting quantified image intensity as mean ± SD (*n* = 5). * *p* < 0.05, vs. DNBS control. ^#^ *p* < 0.05, vs. PBN. ^##^ *p* < 0.05. NM: not measurable.
