# Supplementary material for: Colon-Specific Delivery of Probenecid Enhances Therapeutic Activity of the Uricosuric Agent Against Rat Colitis
Source: Pharmaceutics. 2025 Nov 11;17(11):1454. doi: 10.3390/pharmaceutics17111454 (PMC12655446; doi:10.3390/pharmaceutics17111454)
Supplement: Supplementary file 1 [file pharmaceutics-17-01454-s001.zip › pharmaceutics-3937049-supplementary.pdf]

Supplementary Material S1. Animal experiments and treatment groups

A

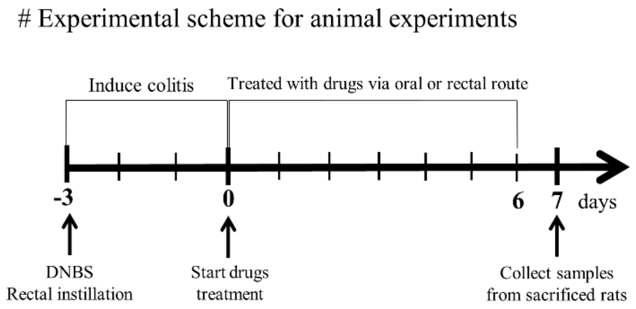

(A) Detailed schemes of animal experiments

(B) Treatment groups in the animal experiments

To evaluate the anticolitic effects of the drugs, the rats were divided into six groups (n =5 per group) and treated as follows: group 1 (normal group), oral gavage of 1.0 mL of PBS; group 2 (colitis group), oral gavage of 1.0 mL of PBS; group 3 (SSZ-treated colitis group), oral gavage of SSZ (50 mg/kg) in 1.0 mL of PBS.; group 4 (PBN-treated colitis group), oral gavage of PBN (30 mg/kg) in 1.0 mL of PBS. group 5 (PBN-GA(L)-treated colitis group), oral gavage of PBN-GA (21.8 mg/kg, equivalent to 15 mg/kg PBN) in 1.0 mL of PBS; group 6 (PBN-GA(H)-treated colitis group), oral gavage of PBN-GA (43.6 mg/kg, equivalent to 30 mg/kg of PBN) in 1.0 mL of PBS.

Supplementary Material S2. Modified scoring system

Score	Feature
0	normal appearance
1	localized hyperemia but no ulcer
2	linear ulcers without significant inflammation
3	2–4 cm site of inflammation and ulceration
4	serosal adhesion to other organs, 2–4 cm site of inflammation and ulceration
5	stricture, serosal adhesion involving several bowel loops, <4 cm site of inflammation and ulceration

Supplementary Material S3. Detailed Protocol for western blot

Tissue samples (0.2 g) were homogenized in 2.0 mL of prechilled radio-immunoprecipitation assay (RIPA) buffer (50 mM Tris-HCl [pH 7.4], 1 mM EDTA, 0.7% Na deoxycholate, 1% NP-40, 150 mM NaCl, 0.3 μM aprotinin, 1 μM pepstatin, and 1 mM phenylmethylsulfonyl fluoride). After agitation on ice for 30 min, the homogenates were subjected to centrifugation at 10,000 × g at 4°C for 10 min. Protein concentrations in the centrifuged lysates were determined using bicinchoninic acid reagent (Thermo Fisher Scientific, Waltham, MA, USA) according to the manufacturer's instructions. Tissue lysates were separated using SDS-PAGE on a 7.5% gel. Cyclooxygenase (COX)-2 and inducible nitric oxide synthase (iNOS) were detected using the following antibodies: anti-COX-2 (sc-365374, Santa Cruz Biotechnology), and anti-iNOS (NOS-2) antibody (sc-7271, Santa Cruz Biotechnology). α-Tubulin (Santa Cruz Biotechnology) was used as a loading control. Western blot images were quantified using Image Lab software (version 5.2 build 14; Bio-Rad, Hercules, CA, USA).

Supplementary Material S4. Full spectra of PBN derivatives

A

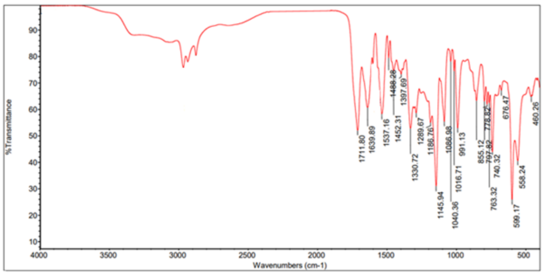

PBN-GA

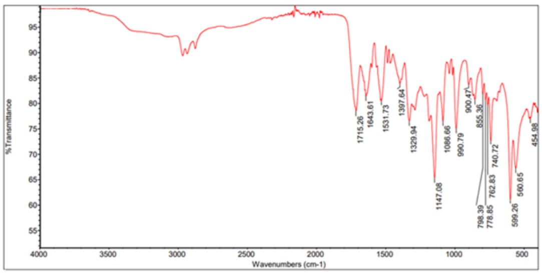

PBN-AA

B

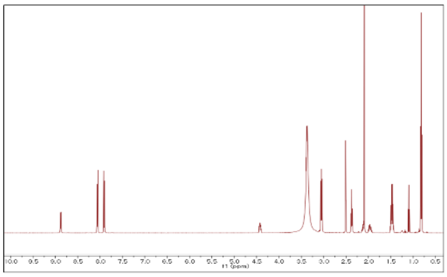

PBN-GA

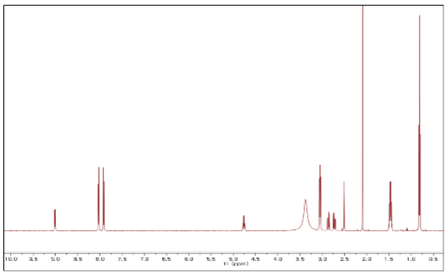

PBN-AA

C

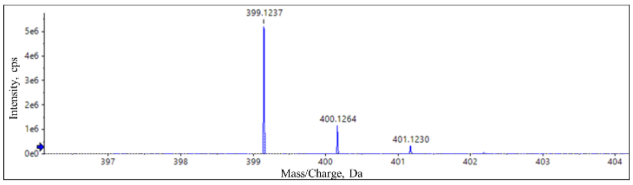

PBN-GA

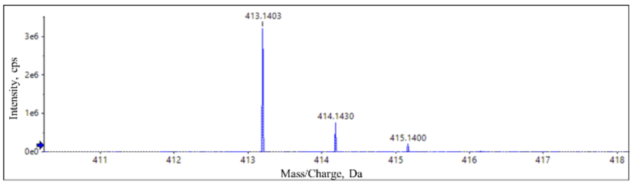

PBN-AA

(A) FT-IR spectra of PBN derivatives

(B) ^{1}H-NMR spectra of PBN derivatives

(C) Mass spectra of PBN derivatives

Supplementary Material S5. Original and quantified images of western blot

A

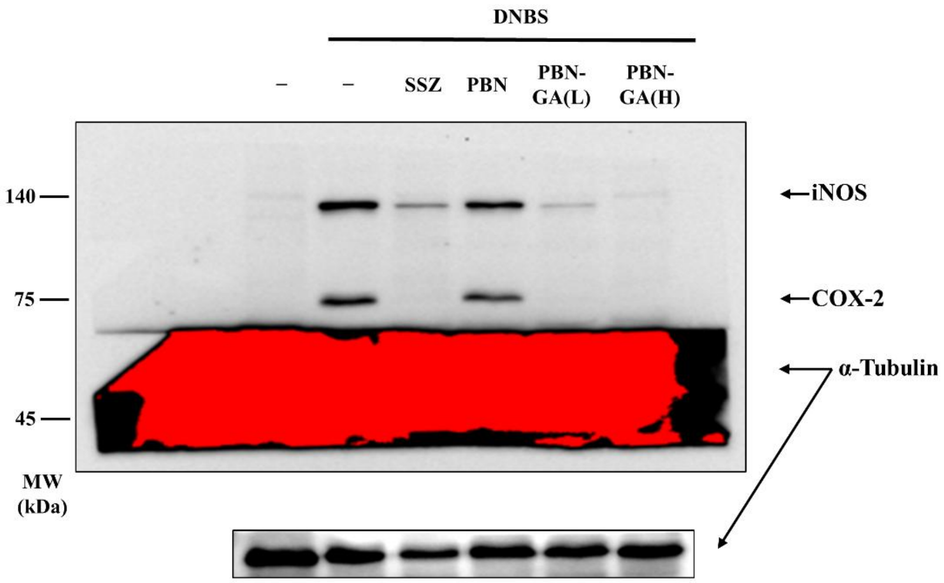

B

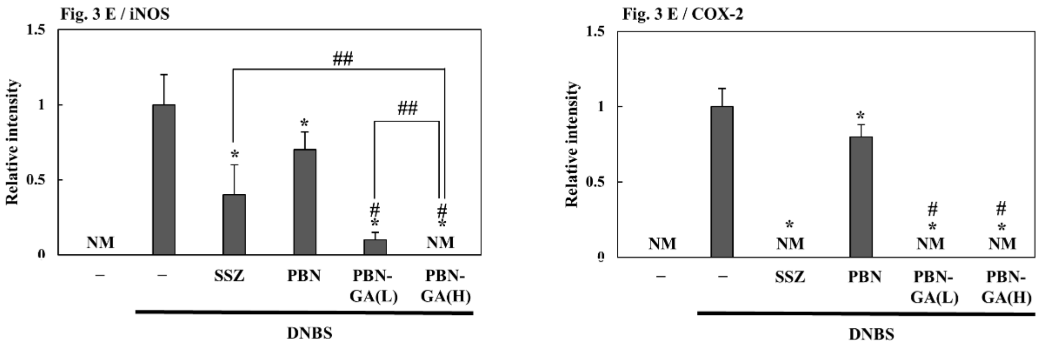

(A) Original images of western blot

(B) Bar graphs presenting quantified image intensity as mean ± SD (n = 5). *p < 0.05, vs. DNBS control. #p < 0.05, vs. PBN. ##p < 0.05, NM: not measurable
